# Neoadjuvant androgen suppression in patients treated with high-intensity focused ultrasound therapy

**Published:** 2007

**Authors:** Makarand V. Khochikar

**Affiliations:** Department of Uro-oncology, Siddhi Vinayak Ganapati Cancer Hospital, Miraj, India. E-mail: khochikar@rocketmail.com

## SUMMARY

The authors have performed this study to explore the effectiveness of neoadjuvant androgen suppression in patients of localized prostate cancer treated with high-intensity focused ultrasound (HIFU). From January 1999 to January 2005, they had treated 250 patients with HIFU. One hundred and fifty-four patients received neoadjuvant hormonal therapy and 96 were not treated with androgen suppression. Treatment failure was defined by presence of prostate cancer on biopsy taken six months after HIFU. In this unrandomized trial, the authors found a slightly lower treatment failure rate in patients who received neoadjuvant androgen suppression (31% vs. 34%), but this was not statistically significant (*P*=0.119).

## COMMENTS

Use of neoadjuvant/concurrent hormonal therapy for localized prostate cancer treated by radical radiotherapy is currently getting studied in many trials. Initial results have been encouraging with significant benefit in the relapse-free /PSA-free interval in these patients.[1] There has been no study published so far studying the use of neoadjuvant androgen suppression in HIFU, this is the first one. The treatment failure was found in 47/154 (31%) patients treated with neoadjuvant androgen suppression as against to 33/96 (34%) patients treated with HIFU only. The difference is not very significant and one could not come to a definitive conclusion from this study. There are many flaws in this study (some of them are pointed out by the authors themselves) such as the nonrandomized design of the study; possible imbalance in the disease severity in both the groups; tendency to treat large-volume higher PSA disease with neoadjuvant androgen suppression as against to more favorable with HIFU alone and interpretation of biopsy after six months of HIFU treatment. Statistically speaking the authors needed 3500 patients in each arm to detect a significant difference of 3% with a power of 80%. Despite all these flaws, the study has been successful in emphasizing the fact that androgen suppression in HIFU does not have any significant side-effects and should be area of research /randomized trials in multimodality treatment of localized prostate cancer.
